# Domain-specific versus general large language models: a review and empirical benchmark in real medical texts

**DOI:** 10.1038/s41598-026-62884-7

**Published:** 2026-07-28

**Authors:** Luís B. Elvas, Carolina Carvalho

**Affiliations:** 1 Department of Logistics, Molde, University College, Britvegen 2, 6410 Molde, Norway; 2 ISTAR, Instituto Universitário de Lisboa (ISCTE-IUL), 1649-026 Lisbon, Portugal; 3 Inov Inesc Inovação – Instituto de Novas Tecnologias, 1000-029 Lisbon, Portugal; 4 Breast Cancer Research Program, Champalimaud Foundation, Lisbon, Portugal

**Keywords:** Named entity recognition, Clinical natural language processing, Large language models, Domain-specific language models, Information extraction, Model efficiency, Energy consumption, Healthcare text mining, Sustainable AI, Computational biology and bioinformatics, Health care, Mathematics and computing

## Abstract

The rapid adoption of Large Language Models (LLMs) has led to their widespread use as a general-purpose, fast-deployment solution across a broad range of tasks. In healthcare settings, this trend has accelerated the replacement of task-specific models with large generative architectures, often without an assessment of their efficiency or suitability for structured information extraction. While transformer-based encoder models have long been established as reliable solutions for clinical Named Entity Recognition (NER) and Information Extraction (IE), the increasing reliance on LLMs raises concerns regarding computational cost, scalability, energy consumption, and practical deployment in resource- and privacy-constrained environments. This study aimed to critically evaluate the assumption that LLMs constitute a universally superior solution for clinical NER and IE, by systematically comparing encoder-based Language Models (LMs), and LLMs in terms of extraction performance, efficiency, deployment feasibility, and environmental impact. Following the Preferred Reporting Items for Systematic Reviews and Meta-Analyses (PRISMA 2020) guidelines, a scoping literature review was conducted using Scopus and Web of Science, covering peer-reviewed studies published between 2022 and 2025. In addition, a proof-of-concept case study was performed on European Portuguese clinical notes, comparing a domain-adapted encoder model (MediAlbertina-1.5B) with quantized Llama-based LLMs executed locally under identical hardware conditions. Models were evaluated using micro-averaged precision, recall, and F1-score, together with runtime, estimated energy consumption, and CO₂ emissions. The literature review showed that LLMs and transformer-based architectures were the most frequently used even thought encoder-based and domain-adapted models often perform strongly in clinical NER, particularly when recall, structured output, and computational efficiency are important. The empirical case study showed a similar pattern: MediAlbertina-1.5B achieved the highest performance (micro-F1 = 0.430), whereas all evaluated Llama variants obtained markedly lower F1-scores (≤ 0.123) due to systematic under-extraction. Despite their lower performance, the Llama models incurred 23–95 × longer runtimes and one to two orders of magnitude higher estimated energy consumption and CO₂ emissions under the tested local CPU-only setup. In this proof-of-concept benchmark on 20 European Portuguese clinical reports, the domain-adapted MediAlbertina token-classification model achieved higher recall and micro-F1 than the evaluated locally executed, quantized Llama models. The Llama models were also substantially slower and less sustainable, with higher estimated energy use and CO₂ emissions under the tested hardware and prompting configuration. These findings suggest that domain-adapted encoder models may be preferable for similar structured clinical NER settings, although larger and externally validated benchmarks are needed before generalizing to other languages, clinical corpora, model families, or deployment environments.

**Supplementary Information:**

The online version contains supplementary material available at 10.1038/s41598-026-62884-7.

## Introduction

Named Entity Recognition (NER) remains a foundational task in Natural Language Processing (NLP), supporting information retrieval, biomedical text mining, and automated knowledge extraction^1–3^. The progression from early shallow models to transformer-based encoders has significantly enhanced contextual understanding, with BERT marking a major shift through its bidirectional architecture and transfer learning capabilities^3,4^. Successive encoder-based models such as RoBERTa and DeBERTa further improved performance through larger pre-training corpora, optimized hyperparameters, and disentangled attention mechanisms^4–6^. Domain-adapted derivatives including BioBERT and ClinicalBERT consistently outperform statistical systems such as CRF and BiLSTM-CRF in scientific and clinical information extraction (IE)^5,7^.

Despite these advances, NER and broader IE remain costly and labor-intensive, particularly in domains requiring expert annotation such as biomedicine or law^8,9^. Data scarcity further limits progress in low-resource languages such as European Portuguese, where only a small number of specialized models exist to support clinical NLP and information extraction tasks^6^. The emergence of Large Language Models (LLMs), such as GPT, Llama, Claude, and Gemini, has redefined expectations for generalization and reasoning, enabling powerful zero-shot and few-shot performance across domains following instruction tuning^3,10–12^. However, token-level accuracy remains a persistent limitation in clinical and biomedical tasks^1^ and multilingual evaluations show reduced reliability in languages underrepresented in pre-training data^13^.

Alongside accuracy concerns, large-scale deployment has also introduced severe efficiency constraints^9^. Full-parameter fine-tuning of models such as Llama-3.1-8B demands more than 140 GB of GPU memory, placing adaptation beyond the reach of most institutions^9^. A second fundamental challenge is the susceptibility of LLMs to hallucinations, factually incorrect or ungrounded outputs, which persist even under well-crafted prompts. Multi-domain evaluations reveal significant hallucination rates across models, with some architectures, such as DeepSeek-R1-Zero showing steadily higher error density despite operating within retrieval-augmented environments^13^. Given the susceptibility of LLMs to hallucinations, Retrieval-Augmented Generation (RAG) has been proposed as a mechanism to ground model outputs in retrieved evidence, thereby reducing factual errors, although at the cost of additional infrastructure for retrieval, indexing, and storage^13^. Other mitigation strategies, such as dual-model agreement or repeated inference, can reduce low-frequency hallucinations but require additional inference cycles and therefore greater computational expenditure^14^. Hybrid pipelines that rerank only the most challenging samples lower hallucination-induced false positives and reduce cost by 80–90%, but they still depend on selectively invoking LLMs, preserving a non-trivial computational footprint^15^.

Amid these challenges, extracting structured information from unstructured clinical text, such as Electronic Health Records, emergency department notes, and biomedical narratives, remains a demanding yet essential task due to dense terminology and irregular linguistic patterns^6^. While LLMs show strong cross-domain generalization and can perform effectively within RAG-based pipelines across Medicine, Agriculture, Biology, Economics, and IoT, their reliability in token-level IE tasks remains uncertain, particularly for low-resource languages such as European Portuguese and Dutch^13,16,17^. Encoder-based architectures such as BioBERT, PubMedBERT, DrLongformer, and MediAlbertina consistently outperform general-purpose LLMs in clinical NER, especially when accurate boundary detection and high recall are required^6,16^. Moreover, RAG-based and large-scale LLM deployments typically rely on cloud-based execution, raising additional concerns regarding data privacy, security, and governance in sensitive clinical settings^17,18^. On the other hand, SLMs can be deployed locally, enabling tighter control over data sharing and compliance requirements^16^. LLMs often exhibit inconsistent token-level predictions, reduced recall, and boundary errors in tasks such as medication extraction, adverse event detection, and clinical concept identification^14,16,19^. Furthermore, optimal strategies for adapting LLMs to domain-specific IE, whether via instruction tuning, few-shot prompting, RAG, or PEFT/QLoRA, remain unclear, and guidance on sample-size requirements for LLM fine-tuning is limited and inconsistent^9^.

Beyond computational and financial cost, the large-scale deployment of LLMs also entails a non-negligible environmental impact^20^. Training and inference of large generative models are associated with substantial energy consumption and carbon emissions, particularly when deployed in cloud-based infrastructures^20^, or when mitigation strategies such as RAG, ensemble agreement, or repeated inference are employed^1^. As LMs models are increasingly integrated into routine and high-frequency workflows, including clinical information extraction, these energy demands raise concerns regarding sustainability and environmental responsibility^21^, reinforcing the need to evaluate model architectures not only in terms of accuracy, but also in terms of energy efficiency and ecological footprint^20^.

In light of these limitations, this study formulates the following research question:

**Research Question (RQ):** To what extent are smaller, domain-adapted LMs more suitable than LLMs for clinical NER in realistic healthcare settings, when jointly considering extraction performance and local-deployment efficiency (runtime, energy consumption, and computational cost)?

To address this RQ, this study conducts a comprehensive empirical comparison of LLMs, SLMs, and encoder-based models for structured IE and NER in specialized clinical contexts. Specifically, the study pursues the following objectives:

- O1 Performance: to compare the clinical NER performance of encoder-based LMs, SLMs, and LLMs using precision, recall, and F1-score.
- O2 Efficiency: to evaluate runtime, latency, and memory requirements across model families under identical inference conditions.
- O3 Environmental Impact: to estimate and compare energy consumption and associated carbon emissions during clinical NER inference.
- O4 Deployment Feasibility: to assess the practicality of local versus cloud-based deployment in terms of cost, infrastructure, and scalability.
- O5 Privacy and Governance: to analyze data privacy and governance implications of local execution compared to cloud-dependent LLMs.
- O6 Reliability: to examine extraction robustness, including hallucinations, under-extraction, and boundary errors in clinical text.

Through these objectives, the study evaluates whether generative LLMs can match or surpass the performance of smaller or domain-adapted encoder models, and identifies which optimization strategies, such as prompt engineering, instruction tuning, PEFT/QLoRA, or hybrid SLM–LLM pipelines, yield meaningful improvements^11^. Beyond accuracy, the evaluation explicitly incorporates computational cost, latency, memory usage, and environmental footprint, with particular emphasis on feasibility for deployment in privacy-sensitive hospital settings where resource constraints are pronounced. In particular, this work emphasizes local execution on existing on-premise hardware, avoiding cloud-based data migration that may compromise data integrity, access control, and patient privacy.

## Review methodology

Following the Preferred Reporting Items for Systematic Reviews and Meta-Analyses PRISMA guidelines^22^, this chapter outlines the methodological framework used to conduct the literature review on LMs applied to clinical NER and structured information extraction. The PRISMA methodology provides a transparent and standardized procedure for documenting search strategies, screening processes, and eligibility criteria, ensuring reproducibility, minimizing selection bias, and enhancing methodological rigor in a rapidly evolving research field.

Within this methodological framework, the review aims to analyze and compare transformer-based model architectures and their application to structured IE and clinical NER. Specifically, it examines the evolution and comparative performance of three major model categories: encoder-only models (such as BERT, RoBERTa, DeBERTa and domain-specific variants including BioBERT and ClinicalBERT), compact and resource-efficient SLMs (e.g., Phi-3-Mini, Llama 3.2–3B), and large generative architectures (including GPT-4, Llama 3.1–8B, Gemini, and MedLM). Through this comparative analysis, the review evaluates trade-offs between predictive accuracy, parameter efficiency, inference latency, memory footprint, scalability under resource constraints, and adaptability to specialized domains, including clinical, biomedical, and multilingual NLP.

The inclusion of peer-reviewed sources from Scopus^23^ and Web of Science^24^ provides an inherent baseline of scientific validity, while the incorporation of high-quality technical reports ensures that cutting-edge developments are not overlooked in this fast-moving research area.

Given the methodological heterogeneity of the included studies and the scoping nature of this review, a formal risk-of-bias assessment was not conducted. This decision is consistent with methodological guidance for scoping reviews, which states that assessments of methodological limitations or risk of bias are generally not performed unless required by the specific aim of the review^25^. Accordingly, the present review was designed to map and describe the available evidence on model performance, efficiency, and deployment feasibility, rather than to produce a critically appraised effect estimate or practice recommendation. Nevertheless, the included studies were interpreted with attention to NLP-specific reporting and reproducibility factors, including dataset transparency, train/test separation or leakage control, metric reporting, prompt or fine-tuning disclosure, model versioning, hardware and runtime reporting, and availability of implementation details. These factors were used to contextualize the strength and comparability of the evidence rather than to exclude studies quantitatively.

This approach is consistent with established practices in computational linguistics and medical informatics, where empirical rigor, dataset transparency, and experimental reproducibility constitute the principal criteria for assessing the validity of NLP research.

### Search strategy

The final database searches were conducted on November 17, 2025, in Scopus and Web of Science Core Collection, using a multi-source strategy aligned with the PRISMA 2020 guidelines^22^. The search strategy was first developed using broad population-level terms and was then progressively refined by adding task-specific and evaluation-related terms. The final Scopus and Web of Science queries were adapted to the syntax of each database and are reported below to ensure transparency, reproducibility, and comprehensive coverage.

The formal peer-reviewed evidence base was built from Scopus and Web of Science. These databases were used for the structured search, eligibility filtering, and final study selection process. Complementary sources, including PubMed^26^, arXiv^27^, ACL Anthology^28^, IEEE Xplore^29^, OpenAI documentation^30^, Meta AI documentation^31^, HuggingFace model cards^32^, Ollama documentation, and other official model documentation, were consulted only to contextualize recent technical developments, model specifications, implementation details, and rapidly evolving methods such as LoRA, QLoRA, quantization, and prompt-based deployment. This combined strategy was designed to balance methodological rigor with timely coverage of recent advances, while keeping the core peer-reviewed evidence base separate from complementary technical sources and acknowledging the rapid development cycle of language modelling research.

The objective of the search was to identify original research articles that empirically evaluated, compared, or optimized the performance and computational efficiency of encoder-based LMs, SLMs, and LLMs in tasks related to NER and IE.

The search was restricted to English-language publications to maintain terminological consistency across studies and ensure the interpretability of technical constructs. Only peer-reviewed journal articles were included in the core dataset, as these undergo accurate methodological scrutiny and provide complete experimental and computational details. Conference papers, workshop abstracts, and preprints were excluded from the final selection due to common limitations in methodological transparency, reproducibility, or completeness of evaluation.

The publication period was limited to 2022–2025, corresponding to the period of accelerated development in transformer-based architectures and instruction-tuned LLMs. This timeframe was selected because it captures the emergence of instruction-tuned and generative LLMs, such as GPT-3, GPT-4, and Llama-3, and the corresponding wave of comparative studies evaluating their efficiency and accuracy against smaller, domain-adapted models (e.g., BERT, DeBERTa, ClinicalBERT, MediBioDeBERTa). Restricting the scope to recent years ensures that the review focuses on current methodologies and architectures that remain relevant to present-day research and deployment scenarios.

### Inclusion Criteria

The inclusion criteria were defined a priori to ensure methodological transparency, reproducibility, and alignment with the objectives of this scoping review. All records included in the formal screening and final synthesis were retrieved from Scopus and Web of Science, using a structured set of Boolean search queries designed to identify peer-reviewed studies evaluating the performance and computational efficiency of LMs applied to NER and IE tasks. Technical repositories, preprint servers, model cards, and official documentation were used only as complementary sources to contextualize emerging methods, implementation details, and model specifications, and were not counted among the 47 studies included in the formal peer-reviewed synthesis.

To guarantee coherence between the research question and the evidence-collection procedure, the screening process was organized according to the Population–Concept–Context (PCC) analytical framework (Table 1). This framework was selected because it provides a clear operationalization of what is being studied (*Population*), which mechanisms or tasks are under investigation (*Concepts*), and under which evaluative or experimental conditions the studies are conducted (*Context*). Applying PCC ensured that the search strategy remained both comprehensive and methodologically aligned with the review’s aim of assessing the performance and efficiency of language models.

**Table 1:** PCC framework and search limitation criteria.

Population	Concepts	Context	Limitations
Language models	Named Entity Recognition	Model Evaluation	Date: 2022–2025
Large language models	Information Extraction	Efficiency Comparison	Subject Area: All
Small language models	Information Structuring	Model Performance	Document Type: Article
Natural language processing		Model Efficiency	
**434 972 Documents**			
	**19 961 Documents**		
		**483 Documents**	
			**192 Documents**

Within the Population dimension, the review included studies examining LMs, SLMs, LLMs and broader NLP systems. These categories encompass the full spectrum of contemporary neural architectures, ranging from encoder-based models such as BERT, RoBERTa and DeBERTa to instruction-tuned and generative LLMs such as GPT, Llama and Gemini.

The Concepts category specified the technical tasks necessary for empirical evaluation: NER, IE, and Information Structuring. These tasks are central to text-mining workflows and constitute the primary mechanisms through which unstructured textual data are transformed into structured, machine-readable information, an essential step for clinical, biomedical, and industrial applications.

The Context criteria further narrowed the scope to studies explicitly addressing Model Evaluation, Efficiency Comparison, Model Performance, or Model Efficiency. This ensured that included publications provided quantitative and reproducible evaluation metrics, such as F1-score, precision, recall, inference latency, throughput, or computational cost, rather than purely theoretical analyses or descriptive accounts lacking empirical validation.

Finally, Limitation criteria were applied to preserve methodological rigor and topical relevance. The publication period was restricted to 2022–2025, capturing the rapid evolution phase of transformer-based and large-scale generative models, including the emergence of instruction-tuned models such as GPT-3.5, GPT-4, Llama-2 and Llama-3. Only peer-reviewed journal articles were included in the final dataset to ensure experimental robustness and methodological transparency, while the subject area remained unrestricted, allowing the inclusion of interdisciplinary research spanning computer science, biomedical informatics, engineering, and applied AI domains. Conference papers, workshop abstracts and preprints were used for manual cross-checking but excluded from the core dataset due to inconsistent reporting standards. When cited, preprints were used only as supplementary contextual evidence for recent technical developments not yet available in peer-reviewed form, and not as records contributing to the PRISMA count or final set of 47 included studies.

The numerical progression reported in Table 1 represents a stepwise search-refinement process rather than successive PRISMA screening stages. The initial broad query, based on population-level terms related to language models and NLP, returned 434,972 database documents. Adding task-specific terms related to Named Entity Recognition, information extraction, and information structuring reduced the results to 19,961 documents. Adding evaluation-focused terms related to model evaluation, model performance, model efficiency, and efficiency comparison further reduced the results to 483 documents. Finally, applying the predefined limits on publication year, document type, and peer-reviewed article status resulted in 192 records selected for formal screening.

Accordingly, the final query used in the Scopus database was defined as follows:

(“Language Models” OR “LMs” OR "Large Language Models" OR “LLMs” OR “SLM” OR “Small Language Models” OR "Natural Language Processing" OR “NLP”).

AND (“NER” OR "Named Entity Recognition" OR “information extraction” OR “information structuring”).

AND (“Model evaluation” OR “Efficiency comparison” OR “Model efficiency” OR “Model performance”).

For Web of Science Core Collection, the equivalent query was**:**

“Language Models” OR “LMs” OR "Large Language Models" OR “LLMs” OR “SLM” OR “Small Language Models” OR "Natural Language Processing" OR “NLP” (Topic) and “NER” OR "Named Entity Recognition" OR “information extraction” OR “information structuring” (All Fields) and “Model evaluation” OR “Efficiency comparison” OR “Model efficiency” OR “Model performance” (All Fields).

### Results

This chapter presents the main findings of the literature search and selection process, detailing the final composition of the studies included in the review. Following the PRISMA 2020 guidelines, a structured, multi-stage screening workflow was implemented to ensure methodological rigor, transparency, and reproducibility.

**Fig. 1:**
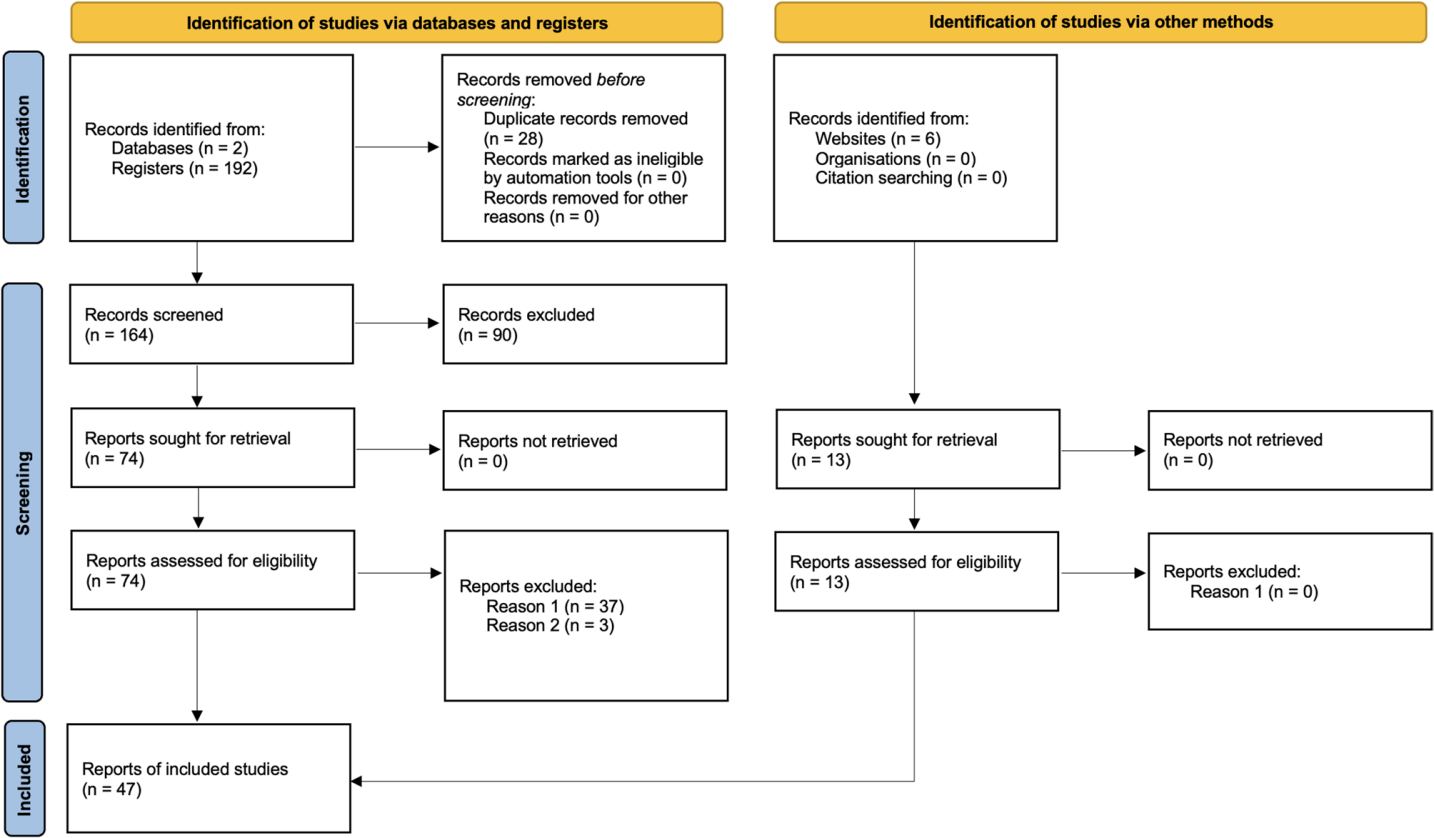
PRISMA flow diagram with the state-of-the-art results.

As illustrated in Fig. 1, the formal PRISMA screening process began with 192 peer-reviewed records retrieved from Scopus and Web of Science after application of the final search query and eligibility limits. Before screening, 28 duplicate records were removed, leaving 164 unique records for title and abstract screening. During this screening stage, 90 records were excluded because they were not sufficiently aligned with the objectives of the review, namely the empirical evaluation of language models for NER, information extraction, or model-efficiency comparison. The remaining 74 reports were retrieved and assessed for full-text eligibility. At this stage, 40 reports were excluded: 37 because they did not address the research objectives in sufficient methodological or empirical depth, and 3 because the full text was not accessible despite institutional and open-access searches. Therefore, 47 peer-reviewed studies met all eligibility criteria and were included in the final synthesis.

The included studies were conducted across diverse linguistic contexts, as summarized in Table 2. English-language contexts were the most common, representing 40.4% of the studies, followed by Chinese (10.6%), French (6.4%), and German (4.3%). Other linguistic contexts were represented in isolated cases, including Spanish, Dutch, Portuguese (PT–PT and PT-BR), Greek, Pashto, Korean, and Luxembourgish, each accounting for 2.1% of the studies. Overall, this distribution highlights the increasing consideration of multilingual settings, while English remains the predominant language context in which clinical and biomedical NLP studies are conducted.

**Table 2:** Linguistic contexts of the studies.

Language	References	Count	Percentage (%)
English	^1,2,7,10–15,20,33–41^	19	40.4
Chinese	^3,42–45^	5	10.6
Spanish	^46^	1	2.1
French	^47–49^	3	6.4
German	^18,49^	2	4.3
Dutch	^17^	1	2.1
Portuguese (PT–PT)	^6^	1	2.1
Portuguese (PT-BR)	^8^	1	2.1
Greek	^50^	1	2.1
Pashto	^51^	1	2.1
Korean	^16^	1	2.1
Luxembourgish	^49^	1	2.1

A methodological overview of the selected studies is presented in Table 3, summarizing model architectures and learning strategies. Transformer-based architectures dominated the literature (89.4%), closely followed using large or pre-trained language models (LLMs/PLMs) (87.2%) and BERT-based models (83.0%). NER was the most common task, addressed in 72.3% of the studies. GPT-based generative models appeared in 55.3% of the works, while prompt learning techniques were used in 46.8%, reflecting growing interest in lightweight adaptation strategies. Other approaches, including Llama-based models (34.0%), few-shot and zero-shot learning (25.5%), and parameter-efficient fine-tuning methods (23.4%), were less frequent but indicate increasing methodological diversification.

**Table 3:** Overview of model architectures and NLP techniques across the reviewed studies.

Techniques	References	Count	Percentage (%)
Transformer architecture/attention mechanism	^1–13,15–20,33–43,46–57^	42	89.4
Large / pre-trained language models (LLMs / PLMs)	^1–5,8–21,33,34,36–39,41–56^	41	87.2
BERT (and variants)	^1–13,16,18–20,32,34–43,45–55^	39	83.0
Named entity recognition (NER)	^1,3–9,11–13,15–19,33,35–39,42–47,50,51,53–56^	34	72.3
GPT based models (GPT-3, ChatGPT, GPT-4, etc.)	^1–4,8,10–15,18,19,34,36–38,42,43,46–48,51,52,54,55^	26	55.3
Prompt learning / prompt engineering	^1,3,8,10–12,14–19,34,35,37,39,43–45,48,49,52^	22	46.8
Llama models	^1,9,11–13,16–18,21,35,37,38,47,49,52,57^	16	34.0
Few-shot learning (FSL)	^3,8–11,15,19,36,43,44,46,52^	12	25.5
CRF (Conditional random field)	^3,5,7,8,36,39,40,42,43,46,50,51^	12	25.5
Zero-shot learning (ZSL)	^1,10,11,16,17,19,34–36,38,39,48^	12	25.5
PEFT (LoRA/QLoRA/Adapter)	^1,9,11,21,37,38,43,44,47,49,54^	11	23.4
Transfer learning / domain adaptation	^1,3,4,6,9,33,37–39,46^	10	21.3
LSTM/BiLSTM/RNN/GRU	^2,3,7,8,36,40,44,50,51^	9	19.1
Graph neural networks (GNN/GCN/GAT)	^1–3,35,45^	5	10.6
Retrieval-augmented generation (RAG)	^1,11,13,17^	4	8.5
Weak supervision learning (WS)	^8^	1	2.1

In summary, the final corpus of 47 studies provides a broad and representative overview of contemporary NLP research, spanning clinical, biomedical, legal, industrial, and multilingual domains. The combined evidence offers a solid empirical foundation for examining the trade-offs between encoder-based models, small language models, and large generative LLMs with respect to accuracy, computational efficiency, domain specialization, and linguistic adaptability.

### Literature review and theoretical background

#### Language models

Several studies evaluated the performance^5–7,10,18,39^, efficiency, and resource demands of traditional LMs across a range of clinical and scientific NLP tasks. These works examined transformer-based encoders, hybrid CRF-based architectures, and domain-adapted biomedical models, focusing on metrics such as precision, recall, and F1-score to quantify their effectiveness. Collectively, the studies explored prompt-based optimization, domain-specific pretraining, long-sequence processing, and supervised sequence labelling, providing a comprehensive assessment of the strengths and limitations of LMs in medical IE and related applications.

One study^10^ evaluates several transformer-based models: GatorTron, ClinicalBERT, BioGPT, and ClinicalT5 using Precision, Recall, and F1-score as primary metrics. Macro-F1 was employed for hyperparameter optimization in multiclass tasks, and GatorTron achieved the highest overall F1-score of 0.919 in the depression classification task. The proposed KOTI method improved the F1-score by 24.12% for GatorTron and 8.35% for ClinicalBERT in few-shot settings, demonstrating the advantage of keyword-based fine-tuning for underrepresented classes^10^. Training was conducted on a single NVIDIA A100 GPU, with pretrained models limited to 512 input tokens. Hyperparameters, including batch size, learning rate, and epochs, were optimized via random search^10^. In terms of computational efficiency, KOTI enabled competitive accuracy with a single forward pass per note, whereas alternative methods required approximately three passes, significantly increasing computational overhead. GatorTron, with approximately 350 million parameters, was also more parameter-efficient than ClinicalT5 (740 million). Although manual annotation remained costly, requiring medical expertise and about ten minutes per document, the study suggested that keyword selection could mitigate part of the annotation burden^10^.

The authors^6^ presented the first large-scale LM trained specifically for European Portuguese clinical data. The model, MediAlbertina-1.5B, achieved a significant reduction in perplexity from 19.48 (baseline Albertina PT–PT) to 1.63, confirming the benefits of domain adaptation^6^. Fine-tuning results showed improvements of 4–6% in Recall and F1-score over the baseline, with macro-F1 scores of 0.848 for NER and 0.755 for Assertion Status tasks. Training was performed on a Linux Ubuntu 20.04 system with 45 GB RAM, 8 vCPUs, and a single Nvidia A6000 GPU (48 GB VRAM), using 2.6 million Electronic Medical Records (≈ 300 million tokens). The configuration included a batch size of 32 for training, 64 for validation, a learning rate of 1e-5, and a token length limit of 128^6^. MediAlbertina’s architecture is based on Albertina PT–PT, containing approximately 900 million parameters, and the training process lasted around 28 days. Manual annotation of over 45 000 tokens by healthcare professionals represented a substantial human-resource cost due to the specialized medical vocabulary^6^.

One approach^5^ benchmarked multiple transformer-based LMs, including Clinical Longformer, ClinicalBERT, DeBERTa v3, Clinical BigBird, SapBERTa, and LinkBERT using strict and lenient F1 metrics (micro and macro) to evaluate NER and event extraction performance. SapBERTa achieved the best lenient F1-score (0.977), while DeBERTa 512 obtained the top strict F1-score (0.954) and the best macro F1 for event extraction (0.844)^5^. Experiments were standardized across models, with 10 epochs and variable sequence lengths (4096 tokens for Longformer, 256 for BERT and DeBERTa). Batch sizes were adjusted to model size (4 for Longformer, 32 for BERT, 16 for DeBERTa). Long-sequence strategies such as a sliding-window approach (256 tokens) were employed to manage input length constraints^5^. Among the evaluated systems, Clinical BigBird demonstrated strong computational efficiency when processing long sequences, while the overall WisPerMed team consistently ranked in the top 10 across challenge categories. The dataset (CMED) was manually annotated by three annotators under medical supervision, underscoring the high cost of labeled data^5^.

A representative study^7^ evaluates three supervised multi-label sequence classification systems, such as CRF, BiLSTM + CRF, and BioBERT + CRF for identifying 24 experimental parameter entities across the Animal, Dose, Exposure, and Endpoint categories in the TAC SRIE 2018 dataset, using token-level and mention-level precision, recall, and F1-score as evaluation metrics computed under strict and lenient matching schemes with ten-fold cross-validation^7^ (Extracting experimental parameter entities from scientific articles). Macro- and micro-averaged precision, recall, and F1 are reported at both category and system level, showing that the CRF model attains higher overall micro and macro precision, whereas the BioBERT + CRF model achieves higher overall micro and macro recall and F1, with system-level micro scores of 0.830 (precision), 0.747 (recall), and 0.786 (F1) and macro scores of 0.834, 0.802, and 0.816 (Extracting experimental parameter entities from scientific articles). The authors further analyze confusion matrices under strict matching as well as the impact of lexical diversity, tokenization, non-contiguous mentions, and category-specific modelling on F1 performance, highlighting statistically significant differences between BiLSTM + CRF and BioBERT + CRF for certain entities based on t-tests over fold-level scores^7^.

A recent study^18^ evaluates the performance of several instruction-tuned language models, such as SauerkrautLM-Nemo-12b-Instruct, SauerkrautLM-gemma-2-9b-it, Phi-3.5-mini-instruct, and Llama-3.1-8B-Instruct on German clinical NER, using precision, recall, and F1-score as the principal evaluation metrics^18^. The study establishes inter-annotator agreement as a performance reference, reporting F1-scores of 0.868 for pathology reports and 0.720 for radiology reports. Across all instruction-tuned models, the results show consistently low F1-scores, ranging from 0.178 to 0.228, primarily due to severely reduced recall despite comparatively high precision, indicating difficulty in recovering the full span of clinical entities^18^. These findings suggest that, within the NER formulation adopted in the study, instruction-tuned LMs struggle to align free-text generative outputs with strict entity extraction requirements. The evaluation therefore applies entity-type–level scoring rather than span-level matching, reflecting an adaptation to generative behavior while preserving the standard precision/recall/F1 analytical framework^18^.

This work^39^ provides a supervised baseline comparison that includes two traditional language models: BioClinicalBERT and a CRF-based sequence labelling system. BioClinicalBERT achieves the strongest performance among all supervised methods, with exact-match F1-scores of 0.785 and relaxed-match F1-scores of 0.901 on the MTSamples dataset, as well as exact and relaxed F1-scores of 0.668 and 0.802 on VAERS^39^. In contrast, the CRF baseline shows substantially lower performance, yielding exact-match F1-scores of 0.584 on MTSamples and 0.525 on VAERS^39^. These results highlight the advantage of domain-adapted transformer encoders over statistical sequence models in clinical NER, particularly under strict matching criteria, and provide a robust reference point for assessing model behavior using precision, recall, and F1-score across datasets with distinct linguistic and structural characteristics^39^.

Taken together, these studies demonstrate that traditional LMs continue to offer a strong balance between accuracy, computational efficiency, and domain adaptability in clinical and scientific NLP tasks. Despite the emergence of LLMs, domain-specific encoders, long-sequence transformers, and CRF-based architectures frequently achieve competitive F1-scores while requiring substantially fewer computational resources and less specialized infrastructure. The collective evidence highlights that, for structured token-level extraction and resource-constrained environments, traditional LMs remain a reliable and cost-effective solution, sustaining their relevance within modern clinical NLP pipelines.

**Table:** 

Model	Architecture/type	Language	Main Task(s)	Best reported metric	Parameters (≈)	Hardware Used	References
BioClinicalBERT	BERT-Based (Encoder)	English	Clinical NER (MTSamples: concepts; VAERS: adverse events)	F1 = 0.785 (MTSamples, Exact Match); F1 = 0.901 (MTSamples, Relaxed Match); F1 = 0.802 (VAERS, Relaxed Match)	–	–	^39^
CRF	Statistical Sequential Tagging	English	Experimental Parameter NER (TAC SRIE 2018); Clinical NER (MTSamples and VAERS)	Macro F1 = 0.616 (System, Lenient)	–	–	^39^
BiLSTM + CRF	RNN-Based Sequential Model	English	Experimental Parameter NER (TAC SRIE 2018)	Micro F1 = 0.578 (System, Lenient) Macro F1 = 0.740 (System, Lenient)	–	–	^7^
BioBERT + CRF	BERT-Based (Encoder) + CRF	English	Experimental Parameter NER (TAC SRIE 2018)	Micro F1 = 0.620 (System, Strict) Macro F1 = 0.711 (System, Strict)	–	–	^7^
gbert-base	BERT-Based (Encoder)	German	Flat NER and Nested NER (Pathology/Radiology Reports)	F1 = 0.874 (Flat NER, Pathology)	111 M	–	^18^
GBERT-BioM-Translation-base	BERT-Based (Encoder, Domain-Adapted)	German	Flat NER and Nested NER (Pathology/Radiology Reports)	F1 = 0.771 (Flat NER, Radiology) F1 = 0.773 (Nested NER, Radiology)	111 M	–	^18^
medBERT.de	BERT-Based (Encoder, Domain-Adapted)	German	Flat NER and Nested NER (Pathology/Radiology Reports)	F1 = 0.778 (Flat NER, Radiology)	109 M		^18^
multilingual-bert-cased	BERT-Based (Encoder, Multilingual)	Multilingual	Flat NER and Nested NER (Pathology/Radiology Reports)	F1 = 0.868 (Flat NER, Pathology)	179 M		^18^
MediAlbertina	DeBERTaV2-based (Encoder)	Portuguese (PT–PT)	NER, Assertion Status	F1 = 0.848 (NER, Multiple Models); F1 = 0.755 (Assertion Status)	900 M	NVIDIA A6000 (48 GB VRAM)	^6^
Albertina PT–PT 900 M	DeBERTaV2 (Encoder)	Portuguese (PT–PT)	NER, Assertion Status	F1 = 0.811 (NER, Multiple Models); F1 = 0.687 (Assertion Status)	900 M	–	^6^
BERTimbau	BERT-Based (Encoder)	Portuguese (PT-BR)	NER, Assertion Status (Comparison)	F1 = 0.789 (NER, Multiple Models)	–	–	^6^
BioBERTpt	BERT-Based (Encoder)	Portuguese (PT-BR)	NER, Assertion Status (Comparison)	F1 = 0.708 (NER, Multiple Models)	~ 110 M		^6^
ClinicalBERT	BERT Variant (Encoder)	English	Classification (KOTI), NER	Lenient F1 = 0.962 (NER) Relaxed Match F1 = 0.901 (NER)	–	NVIDIA A100 GPU	^5,10,39^
GatorTron	Transformer (Encoder)	–	Clinical Note Classification (KOTI)	F1 = 0.919 (Depression, KOTI)	~ 350 M	NVIDIA A100 GPU	^10^
Clinical Longformer	Longformer-based (Long-sequence Encoder)	English	NER, Event Classification	Lenient F1 = 0.965 Micro F1 = 0.892 (NER + Event)	–	–	^5^
DeBERTa v3 (512)	DeBERTaV3 (Encoder)	English	NER, Event Classification	Strict F1 = 0.954 Macro F1 = 0.844	–	–	^5^
Clinical BigBird	Transformer (Encoder)	English	NER, Medication Extraction	Lenient F1 = 0.971	–	–	^5^
SapBERT	Transformer (Encoder)	English	NER, Medication Extraction	Lenient F1 = 0.977	–	–	^5^
LinkBERT	Transformer (Encoder)	–	NER, Event Classification	Micro F1 = 0.914 Macro F1 = 0.814	–	–	^5^
ClinicalT5	Transformer T5-based (Encoder-Decoder)	English	Classification (KOTI)	F1 = 0.850	~ 740 M	NVIDIA A100 GPU	^10^
BioGPT	Transformer (Decoder-Only)	English	Text Generation / Classification	F1 = 0.800	-	NVIDIA A100 GPU	^10^

#### Large language models (LLMs)

LLMs have become central to contemporary research in domain-specific IE, offering strong generative and reasoning capabilities but also introducing substantial computational and resource demands. Recent studies evaluating models such as Llama-3.1-8B, GPT-3, GPT-4, GPT-4 V, and other instruction-tuned or multimodal LLMs illustrate both the potential and the limitations of these systems across biomedical, legal, industrial, and clinical NER settings. Across these works, effectiveness is primarily assessed through Precision, Recall, F1-score, and error analysis, while resource requirements are measured in GPU memory consumption, inference latency, fine-tuning feasibility, and monetary cost. Together, these evaluations provide a comprehensive perspective on how LLMs behave under realistic constraints, and how their scalability, performance, and operational overhead compare to more compact alternative models.

Recent work on parameter-efficient adaptation of LLMs^9,11^ demonstrate the central role of parameter-efficient optimization techniques in improving the feasibility and performance of LLMs for specialized IE tasks. One work^9^ investigated the adaptation of Llama 3.1-8B-Instruct using QLoRA and showed that the resulting model, NERLlama3.1, achieved high micro-F1 scores of 0.8977 (NCBI-disease), 0.9402 (BC5CDR-chemical), and 0.8530 (BC2GM-gene), outperforming both fully fine-tuned LLM baselines and BioBERT, with an average F1 of 0.8970 across datasets and strong generalization to unseen corpora (mean F1 = 0.7351)^9^. The study further demonstrated that QLoRA substantially reduces computational requirements: while full fine-tuning of the 8-billion-parameter model demands more than 140 GB of GPU VRAM, QLoRA lowers training memory usage to 12.6 GB (r = 32) or 13.3 GB (r = 64) and inference memory to 6 GB, enabling 24-h training on a single NVIDIA 4080 Super GPU and updating only 54.5 million parameters rather than the full model^9^. Complementing these findings, Vithanage et al.^11^ evaluated Llama 3.1-8B across zero-shot, few-shot, PEFT-LoRA, and RAG configurations, showing that in-context examples substantially improve performance over zero-shot (+ 17% Accuracy, + 17% Precision, + 23% Recall, + 19% F1)^11^. PEFT (LoRA) provided further gains, increasing zero-shot performance by + 26% Accuracy, + 33% Precision, + 31% Recall, and + 29% F1, while also enhancing few-shot results (+ 16% Accuracy, + 11% Precision, + 14% Recall, + 18% F1)^11^. Although RAG did not benefit zero-shot settings, it significantly boosted few-shot performance (+ 19% Accuracy, + 15% Precision, + 16% Recall, + 21% F1) when contextual exemplars were available^11^. In terms of resource usage, PEFT experiments required four NVIDIA RTX-A5000 GPUs (24 GB each), while prompt-based and RAG configurations ran on a single 24 GB GPU, illustrating that PEFT reduces the proportion of trainable parameters to below 0.01% and thereby offers a more resource-efficient alternative to full fine-tuning^11^. Together, these studies underscore that both QLoRA and LoRA provide robust and computationally accessible pathways for adapting LLMs such as Llama 3.1-8B to domain-specific IE tasks, delivering substantial performance improvements while constraining memory and compute demands.

Several studies^8,39^ highlight the role of prompt-based LLMs in reducing annotation costs while revealing clear performance and resource trade-offs across legal and clinical NER tasks. A comparative study^8^ evaluated compared human annotation, GPT-3–based labeling, and weak supervision pipelines, showing that manual annotation yielded the highest F1 performance (0.713 across all entities; 0.877 for the top seven), while weak supervision achieved competitive scores (0.679 overall; 0.873 for top entities) at substantially lower cost^8^. GPT-3 alone lagged behind (0.528 overall; 0.763 for top entities), but hybrid methods demonstrated strong retention of performance: weak supervision achieved the highest Preservation Score (PS = 0.956 for all entities, 0.996 for top entities), followed by GPT + weak supervision (0.907 / 0.963) and GPT + 30% human input (0.839 / 0.935), whereas GPT-3 alone had the lowest preservation (0.740 / 0.867)^8^. The study also quantified labeling costs, reporting that GPT-3 (Davinci) required $0.02 per 1000 tokens, totaling $31.30 for 1.56 million tokens across 783 documents, while relying on pretrained encoders such as BERTimbau, Lener-BR, RoBERTa, and DistilBERT-PT with a BI-LSTM layer for supervised baselines^8^. Complementing these findings, a recent study^39^ evaluated GPT-3.5 and GPT-4 for clinical concept extraction and found that baseline relaxed-match F1-scores were modest for both models (GPT-3.5: 0.634 on MTSamples, 0.301 on VAERS; GPT-4: 0.804 on MTSamples, 0.593 on VAERS)^39^. Prompt-engineering strategies, combining annotation-guideline cues, error-analysis instructions, and few-shot examples, substantially improved performance, enabling GPT-4 to reach relaxed F1 values of 0.861 on MTSamples and 0.736 on VAERS^39^. Despite these gains, LLMs remained inferior to supervised encoder-based baselines, and the study emphasized the financial overhead of token-based APIs, with GPT-3.5 costing $0.03–$0.06 per 1000 tokens and GPT-4 costing $0.001–$0.002, underscoring that computational and monetary costs remain significant barriers to large-scale clinical deployment^39^.

One study^1^ proposed an evaluation pipeline for GPT-4 V and related multimodal LLMs^1^. GPT-4 V achieved an overall accuracy of 84%, answering 66 of 79 medical image-based questions correctly^1^. Performance varied by subdomain, with F1-scores of 1.00 for Neoplasia and Cardiovascular Pathology, and 0.89 for Atherosclerosis, Thrombosis, and Endocrine Pathology^1^. Network graph analysis showed that incorrect responses had a higher graph density (0.0633) and fewer connected components (13), suggesting error clusters amenable to targeted fine-tuning^1^. LLMs such as Mistral and Llama were noted for using tens of billions of parameters, making large-scale fine-tuning resource intensive. Multimodal training across image, audio, and video data was identified as particularly costly. The framework emphasized Parameter-Efficient Fine-Tuning (PEFT, LoRA adapters) as a means to reduce hardware requirements and computational burden by updating only a subset of parameters^1^. The X-REQ approach was introduced as a cost-saving strategy that avoids full model retraining by focusing fine-tuning on underperforming diagnostic domains. All interactions with GPT-4 V were limited to a maximum of 4 000 tokens^1^

A comparative evaluation^15^ evaluated several contemporary LLMs across nine datasets spanning NER, Relation Extraction, Event Detection, and Event Argument Extraction, finding that LLMs consistently underperformed fine-tuned SLMs in few-shot extraction scenarios, as measured by micro-F1^15^. The authors report that LLMs achieve competitive results only when both the number of labelled samples and the variety of entity types are extremely limited, but performance deteriorates substantially once more representative training samples are available, primarily due to lower Recall^15^. Latency and cost analyses show that LLM inference incurs significantly higher computational expenditure than small models, with increased runtime and token consumption even under identical prompts^15^. Despite these limitations, the study demonstrates that LLMs can be effective rerankers for difficult samples, achieving an average 2.4% F1 improvement when paired with SLM filters in a filter-then-rerank pipeline, at a modest computational overhead^15^.

An industry-oriented study^49^ examined the applicability of LLMs to domain-specific IE in industry settings, highlighting that although LLMs can achieve competitive Precision and F1 in constrained tasks, their deployment incurs substantial memory, compute, and energy costs compared with smaller models^49^. The authors report that LLM-based extraction pipelines suffer from high inference latency and increased monetary cost per request, making them less feasible for high-volume business applications unless paired with optimization techniques such as caching or lightweight retrieval augmentation^49^. Benchmarking further reveals that many extraction tasks do not benefit proportionally from increased model size, with smaller models often matching LLM performance once domain-specific structure and constraints are incorporated^49^. These findings reinforce that compute-efficient alternatives may offer a more favorable balance of accuracy and operational cost in industrial IE settings^49^.

An empirical evaluation^18^ assesses instruction-based LLMs were evaluated for clinical NER, revealing that LLMs consistently achieved high precision but substantially lower F1-scores, ranging from 0.18 to 0.30, due to markedly reduced recall^18^. The results show that the tested LLMs, although capable of generating accurate individual entities, tended to produce fewer outputs overall, suggesting overly conservative extraction behaviour in multi-entity clinical documents^18^. The study highlights that the computational overhead associated with LLM inference does not yield proportional gains in extraction accuracy, reinforcing that current LLMs remain limited for comprehensive clinical entity extraction tasks involving many entity types per document^18^. The authors note that prompt refinements instructing the models to output more entities may improve recall, but further work is required to close the performance gap relative to encoder-based NER systems^18^.

A size-based comparison^52^ evaluated SLMs and LLMs for news summarization using BERTScore F1 as the principal metric to assess semantic similarity and content preservation^52^. The results show that while larger models can achieve marginally higher BERTScore values, the performance gap between LLMs and smaller models is minimal, with several smaller models performing comparably in factual consistency and relevance^52^. The authors highlight that these improvements come at a substantial computational cost, noting that deploying a 70B-parameter model typically requires multiple high-end GPUs and significantly more memory and energy consumption than smaller models^52^. Furthermore, the study reports that summarization quality does not scale proportionally with model size, as many SLMs are capable of real-time summarization on edge devices, demonstrating diminishing returns relative to the increased hardware requirements of LLMs^52^. Overall, the findings suggest that while LLMs possess strong generative capabilities, smaller models often offer a more efficient balance between summarization performance and computational feasibility in resource-constrained environments^52^.

An empirical analysis^34^ evaluated extraction performance across models of different sizes and found that smaller transformer models achieved relatively accurate structured data extraction, with error rates as low as 10%, while maintaining significantly lower computational requirements than LLMs^34^. The authors report that model size influences the balance between accuracy and compute cost, noting that larger models do not necessarily provide higher extraction yield and may suffer diminishing returns relative to their substantial resource requirements^34^. The study concludes that although LLMs demonstrate strong language understanding, smaller models remain more practical for domain-specific extraction workflows that require efficient deployment and locally controlled compute environments^34^.

A multidimensional evaluation framework^13^*,* assesses the robustness of state-of-the-art LLMs, GPT-4 Turbo, Claude-3.7-Sonnet, LLaMA-3.3-70B, DeepSeek-R1-Zero, and Gemini-2.0-Flash, across five knowledge domains using a composite evaluation scheme integrating semantic similarity, sentiment bias, hallucination detection, and NER-based factual verification^13^. The results show that LLaMA-3.3-70B achieved the highest composite scores across all domains, demonstrating superior robustness and factual reliability under retrieval-augmented evaluation conditions^13^. The framework revealed persistent challenges for all LLMs, including domain-specific hallucinations, semantic drift, and variability in factual grounding, which remained measurable even under controlled retrieval-augmented prompts^13^. The authors emphasize that LLM deployment in high-stakes fields requires multidimensional evaluation beyond fluency, and that hallucination detection and factual verification remain critical limitations of current large-scale generative models^13^.

**Table:** 

Model	Architecture/type	Language	Main task(s)	Best reported metric	Parameters (≈)	Hardware used	References
Llama 3.1-8B (NERLlama3.1)	Decoder (Instruction-tuned via QLoRA)	English	NER	F1 = 0.8977 (NCBI) F1 = 0.9402 (BC5CDR-Chem) F1 = 0.8530 (BC2GM)	8B	NVIDIA 4080 Super (16 GB VRAM)	^9^
Llama 3.1-8B (IE Baseline)	Decoder (Zero-shot, Few-shot, PEFT, RAG)	English	IE	F1 = 0.593 (Exact Match) F1 = 0.861 (Relaxed Match)	8B	4 × NVIDIA RTX-A50000 (24 GB)	^11^
GPT-3 (Davinci)	Decoder (Generative LLM)	PT-BR	Legal NER (Automatic labelling)	F1 = 0.528 (all entities) F1 = 0.763 (top entities)	–	OpenAI API (Cloud)	^8^
GPT-4	Decoder (Generative LLM)	English	Clinical NER (MTSamples, VAERS)	F1 = 0.861 (MTSamples, Relaxed) F1 = 0.736 (VAERS, Relaxed)	–	OpenAI API (Cloud) Token cost $0.001–$0.002/1 k tokens	^39^
GPT-3.5	Decoder (Generative LLM)	English	Clinical NER	F1 = 0.634 (MTSamples, Relaxed) F1 = 0.301 (VAERS, Relaxed)		OpenAI API (Cloud) Token cost $0.03–$0.06/1 k tokens	^39^
GPT-4 V	Multimodal (Vision + Text)	English	Medical Image Question Answering, Diagnostic IE	Accuracy = 84% F1 = 1.00 (Neoplasia) F1 = 0.89 (Other Domains)	–	–	^1^
LLaMA-3.3-70B	Decoder (Generative LLM)	English	Cross-domain generation; Hallucination analysis	Final Score* = 1.629	70B	–	^13^
Claude-3.7-Sonnet	Decoder (Generative LLM)	English	Cross-domain generation; Hallucination detection	Final Score* = 1.183	–	–	^13^
Gemini-2.0-Flash	Decoder (Generative LLM)	English	Cross-domain IE, generation; Hallucination detection	Final Score* = 1.060	–	–	^13^
DeepSeek-R1-Zero	Decoder (Generative LLM)	English	Cross-domain generation; Hallucination detection	Final Score* = 0.686	–	–	^13^
Instruction-tuned LLMs in clinical NER (SauerkrautLM-Nemo-12B, SauerkrautLM-Gemma-2-9B-IT, Phi-3.5-Mini-Instruct, Llama-3.1-8B-Instruct)	Decoder (Instruction-tuned)	German	Clinical NER (Pathology/Radiology)	F1 = 0.18–0.30 (High Precision, Low Recall)	7B–12B	–	^18^

* **The Final Score** represents a composite metric derived through Min–Max and Z-Score normalization of four core evaluation dimensions: semantic similarity, sentiment neutrality, TF-IDF similarity (reflecting factual alignment and aiding hallucination detection), and NER-based precision. It is used to rank the overall performance, robustness, and effectiveness of each LLM across the five evaluated knowledge domains (Agriculture, Biology, Economics, IoT, and Medicine).

#### Efficiency in model scaling

Scaling the size of pre-trained LMs improves representational capacity and enables stronger generalization, but it simultaneously amplifies memory, computation, and energy demands, creating substantial barriers to practical deployment in resource-constrained environments^9^. Full-parameter fine-tuning remains particularly costly: for instance, training an 8-billion-parameter model such as Llama 3.1 requires more than 140 GB of GPU VRAM and incurs significant computational load even for inference, illustrating the inefficiency of conventional training paradigms when applied to large-scale architectures^9^. PEFT methods, including QLoRA, address these limitations by introducing 4-bit quantization and low-rank adaptation layers that reduce VRAM requirements to 12–13 GB during training and approximately 6 GB during inference, enabling 24-h training cycles on a single consumer-grade GPU while maintaining state-of-the-art performance in biomedical NER^9^. These techniques demonstrate that reducing the proportion of trainable parameters does not necessarily compromise accuracy, and that selective updates provide a viable path toward scalable adaptation under hardware constraints^9^.

Beyond fine-tuning, the scaling efficiency of LMs also depends heavily on architectural and operational cost. LLMs exhibit high expressive power but require substantial computational resources for both training and inference, which limits their viability in real-time or edge-based systems and increases operational expenditure for sustained deployment^21^. SLMs, by contrast, offer a more favorable balance between performance and cost, as models under 4B parameters achieve competitive accuracy while reducing FLOPs, energy consumption, and inference latency by up to an order of magnitude when compared to models above 70B parameters^21^. Empirical evidence further shows that SLMs benefit disproportionately from architectural optimization: models such as SlimLM use aggressive 4-bit quantization and compression strategies to operate directly on mobile hardware, reducing reliance on cloud APIs and enabling on-device document assistance without sacrificing practical utility^57^. These findings highlight that efficiency in model scaling is not solely a function of parameter count, but rather of the interplay between architecture, quantization, and the deployment environment^57^.

Efficiency considerations also extend to latency, throughput, and CPU-based inference. Distilled models like TinyGreekNewsBERT reduce computational cost by a factor of 15 × requiring only 1.3 BFLOPs and achieve median CPU latencies of 14.7 ms per document, offering more than a 10 × speedup over larger transformer models while maintaining strong performance in NER and text classification tasks^50^. Such efficiency gains enable large-scale content processing on consumer hardware and illustrate the advantages of scaling down model size for production settings that prioritize speed and resource minimization^50^. Similarly, domain-specific compact architectures such as MechBERT demonstrate that targeted pre-training on specialized corpora can outperform much larger models such as RoBERTa-LARGE and BERT-LARGE while being three times smaller and six times faster, underscoring the principle that relevance and quality of pre-training data often matter more than the raw parameter count for domain-restricted extraction tasks^20^. These results collectively show that reducing model size not only accelerates inference but also lowers energy consumption and improves sustainability, particularly in applications where continuous, high-volume processing is required^20^.

Efficiency in model scaling is further shaped by the quantity of training data required for effective fine-tuning. Contrary to common assumptions that larger models demand extensive datasets, evidence shows that NER performance often saturates rapidly, with diminishing returns beyond a threshold of approximately 439 labelled sentences for models such as RoBERTa-LARGE^56^. Additional experiments demonstrate that architecture exerts greater influence on performance than model size, as the largest model tested (GPT-2_large) delivered the weakest results in specific NER settings despite having the highest parameter count^56^. These findings highlight that efficient data scaling, optimizing the amount and type of data rather than merely expanding quantity, is an essential aspect of cost-effective model scaling and reduces unnecessary annotation effort^56^.

Overall, efficiency in model scaling emerges from a combination of parameter-efficient training, quantization, architectural optimization, and data-efficient fine-tuning. While LLMs offer powerful generalization and reasoning abilities, their computational and financial costs remain substantial, motivating the adoption of SLMs, PEFT methods, and specialized compact architectures as practical alternatives for real-world domain-specific NLP systems^20,21,57^.

#### Empirical comparison of SLMs, LMs, and LLMs

**LLM performance in multi-domain and generative tasks**

LLMs consistently demonstrate strong robustness, semantic coherence, and retrieval-augmented reasoning when evaluated across diverse knowledge domains. In a comprehensive multi-domain framework spanning Agriculture, Biology, Economics, IoT, and Medicine, the LLaMA-3.3-70B-Versatile model achieved the highest composite score among state-of-the-art LLMs, including GPT-4-Turbo, Claude-3.7-Sonnet, Gemini-2.0-Flash, and DeepSeek-R1-Zero, showing superior performance in semantic similarity, factual verification, hallucination resistance, and sentiment neutrality^13^. These results highlight that LLMs excel in tasks requiring broad cross-domain generalization and holistic reasoning, particularly under retrieval-augmented settings that integrate semantic similarity, TF-IDF factual alignment, and NER-based verification into a unified evaluation metric^13^. Collectively, the evidence indicates that, although LLMs may vary in reliability across domains, they display a consistent advantage when the task involves high-level abstraction, multi-domain generality, or composite evaluation criteria rooted in both semantic coherence and factual grounding^13^.

**LLM weaknesses in clinical NER and structured extraction**

Despite their strengths in generative and semantic-level tasks, LLMs consistently underperform encoder-based LMs and SLMs in domain-specific NER, particularly in clinical contexts where recall is critical. In German radiology and pathology NER, encoder-based models such as medBERT.de and GBERT-BioM-Translation-base achieved F1-scores up to 0.778, whereas instruction-tuned LLMs such as SauerkrautLM, Llama-3.1-8B-Instruct, and Phi-3.5-Mini-Instruct produced drastically lower F1-scores between 0.178 and 0.228, primarily due to extremely low recall despite high precision^18^. This pattern extends to general IE tasks with complex label sets: LLMs using in-context learning show steep degradation when label cardinality increases, performing notably worse on datasets such as MAVEN with 168 labels compared to simpler schemes such as CONLL with only four labels^15^. Crucially, supervised SLMs continue to improve performance as sample size increases, whereas LLMs exhibit plateauing behavior, outperforming supervised models only in the strict few-shot regime (1-shot or 5-shot) but falling behind once more annotated data becomes available^15^. Even in multilingual settings, LLMs offer strong zero-shot NER performance across Latin and non-Latin languages, yet encoder-based multilingual LMs such as XLM-R remain superior when supervision is available, achieving 96.24% accuracy in Pashto POS tagging and outperforming RNN/BiLSTM-CRF systems on OOV and homograph classification^35^. These results confirm that LLMs struggle in structured extraction tasks requiring fine-grained span detection, high recall, and stability across dense entity distributions, domains in which encoder-based LMs remain empirically dominant.

**SLMs and domain-specialized LMs as competitive or superior alternatives**

SLMs and domain-specialized encoder-based LMs frequently match or exceed LLM performance while requiring only a fraction of the computational resources. In news summarization, instruction-tuned SLMs such as Phi-3-Mini and Llama-3.2-3B-Instruct achieved semantic relevance, coherence, and factual consistency comparable to 70B-parameter LLMs, while producing more concise summaries and operating with substantially lower inference cost^52^. In domain-specific IE, pre-training on a focused corpus proved more valuable than scaling model size: MechBERT, trained exclusively on mechanical stress and strain literature, outperformed RoBERTa-LARGE and BERT-LARGE on specialized QA benchmarks despite being significantly smaller, highlighting that corpus relevance can outweigh raw parameter count for specialized IE tasks^20^. Likewise, the CLIN-XEN and CLIN-XES models, domain-adapted variants of XLM-R surpassed general-purpose multilingual encoders such as mBERT and XLM-R in English and Spanish clinical NER, demonstrating the utility of domain adaptation for structured clinical extraction^46^. Zero-shot multilingual NER further revealed mixed dynamics: although ChatGPT achieved higher zero-shot NER performance across multiple languages than encoder-based GLiNER, supervised encoder models such as fine-tuned XLM-R remained superior whenever labelled data was available, reinforcing the advantage of encoder architectures for high-recall, label-dense extraction scenarios^35^. Collectively, these findings show that SLMs and domain-specialized encoder-based LMs remain highly competitive and in many cases superior to LLMs in structured, domain-constrained, or label-rich IE settings.

### Effects of fine-tuning, instruction tuning, and hybrid techniques

Fine-tuning and instruction tuning substantially reshape the empirical relationship between SLMs, LMs, and LLMs in IE settings. In biomedical NER, parameter-efficient instruction tuning enables LLMs to exceed the performance of specialized encoder models: the NERLlama3.1 model, trained with QLoRA, achieved a mean F1-score of 0.8970 across BioNER datasets, statistically surpassing BioBERT and PubMedBERT while requiring only 6–13 GB of GPU memory through 4-bit quantization^9^. Hybrid optimization strategies further enhance extraction performance: a filter-then-rerank pipeline, in which supervised SLMs first filter easy instances and LLMs rerank only the difficult cases, produced an average F1 improvement of 2.4% across NER, RE, ED, and EAE benchmarks, demonstrating that LLM reasoning is most valuable when selectively applied rather than used end-to-end^15^. Prompt design also critically determines LLM effectiveness: in medication-prescription NER and text-expansion tasks, structured prompts incorporating annotation rules and few-shot demonstrations led cutting-edge LLMs such as ChatGPT-4o and Gemini 2.0 Flash to outperform ChatGPT-3.5, reducing hallucinations and substantially increasing extraction accuracy^19^. Taken together, these findings show that although raw, zero-shot LLMs often underperform encoder-based models in structured extraction tasks, domain-specific instruction tuning, PEFT-based fine-tuning, hybrid pipelines, and carefully engineered prompts can enable LLMs to recover or exceed the performance of traditional LMs and SLMs when applied under optimized conditions.

Empirical evidence demonstrates that the performance landscape across SLMs, encoder-based LMs, and LLMs is far from linear or scale-deterministic. Instead, effectiveness in IE hinges on the interaction between model architecture, domain adaptation, supervision regime, and optimization strategy. Encoder-based LMs remain the most reliable choice for structured, high-recall tasks such as clinical NER, where their token-level precision and domain-specific pre-training systematically outperform instruction-tuned LLMs. SLMs, despite their smaller parameter footprint, achieve competitive results in generative tasks such as news summarization and often surpass larger models when domain constraints, efficiency requirements, or real-time deployment conditions are present. Conversely, LLMs excel in multi-domain reasoning, retrieval-augmented generation, and zero-shot generalization, yet exhibit characteristic weaknesses in label-dense extraction tasks due to low recall and inconsistent boundary detection.

Across all architectures, performance disparities narrow substantially when fine-tuning, PEFT techniques, hybrid filtering-and-reranking pipelines, or advanced prompt design are introduced. These strategies reveal that LLM underperformance in structured extraction is not intrinsic to scale but largely a consequence of unadopted use. At the same time, specialized encoders and SLMs continue to offer a more favorable accuracy-to-cost ratio for domain-specific IE, particularly in low-resource or high-volume settings. Collectively, these findings highlight that no single model family dominates universally; rather, optimal performance arises from aligning model class with task structure, domain specificity, and available computational resources, underscoring the need for adaptive, context-aware model selection in practical IE pipelines.

## Proof-of-concept: a case study comparing fine-tuned clinical LM vs. llama-based LLMs

This case study examines the comparative behavior of five open-source LMs applied to clinical NER in European Portuguese. The goal is to characterize how different model families, domain-specific encoder architectures, SLMs, and larger instruction-tuned LLMs, perform when exposed to the same clinical extraction task and operational constraints.

The evaluation includes the domain-adapted encoder-based model MediAlbertina-1.5B, two SLMs (Llama 3.2-3B and Llama 3.2-3B-Instruct, executed in Q6_K quantization), and two larger models from the same family (Llama 3.1-8B and Llama 3.1-8B-Instruct, executed in Q4_0 quantization). Encoder-based models are applied through an existing NER-fine-tuned checkpoint, while all Llama variants are run locally via Ollama using structured prompting, a PT–PT clinical schema, and few-shot examples. This setup reflects realistic deployment conditions, in which quantized LLMs are required to operate within standard hardware constraints typically found in clinical research settings.

All models are assessed on the same corpus of de-identified Portuguese clinical notes, with outputs normalized to a shared set of entity types and character-level spans. By placing encoder models, quantized SLMs, and quantized LLMs under a unified evaluation framework, this case study aims to investigate how model architecture, scale, and instruction tuning influence their suitability for clinical IE pipelines in Portuguese, and to provide a structured basis for the comparative analysis presented in the subsequent sections.

### Methodology

The dataset consists of 20 real clinical reports in Portuguese (PT–PT), collected from Hospital de Santa Maria the largest public hospital in Portugal, and exported in CSV format (file *relatorios_20.csv*). The data were provided under the framework of the FCT-funded project DSAIPA/AI/0122/2020 (AIMHealth—Mobile Applications Based on Artificial Intelligence)^58^, with database availability for research approved by the Ethical Committee of the Faculty of Medicine of Lisbon on February 17, 2023 (reference number 132/22). This study used retrospective, de-identified text reports from clinical examinations and did not involve any direct interaction or intervention with human participants. All methods were performed in accordance with the relevant guidelines and regulations. Due to the retrospective nature of the study, the Ethical Committee of the Faculty of Medicine of Lisbon waived the need to obtain informed consent. The field DIARIO_CLINICO contains the free-text clinical narrative, while the first column serves as a unique identifier for each document. On average, each line contains approximately 36.65 characters, reflecting short, telegraphic clinical phrasing typical of inpatient notes. All gold-standard annotations were manually produced by an oncology specialist affiliated with the Champalimaud Foundation, following the predefined eight-class clinical NER schema used in this study. The annotation schema includes eight clinically relevant entity types: *Diagnosis*, *Symptom*, *Medication*, *Dosage*, *MedicalProcedure*, *VitalSigns*, *Result*, and *Progress.*

For each document, gold entities are represented as a list of objects containing the surface text, entity type, and start–end character positions relative to the original note.

Five models were evaluated in a deployment-oriented setup: one domain-adapted, NER-specific encoder checkpoint and four locally executed, quantized Llama-based generative models used through prompting. The encoder baseline was MediAlbertina-1.5B, accessed through a NER-fine-tuned HuggingFace token-classification checkpoint. In contrast, the Llama models were not task-specific NER checkpoints, they were evaluated through structured prompting, a predefined PT–PT clinical entity schema, and few-shot examples using Ollama. The encoder baseline is MediAlbertina-1.5B, a Portuguese clinical model accessed via the HuggingFace Transformers framework using the checkpoint portugueseNLP/medialbertina_pt-pt_1.5b_NER. In this setting, tokenization and inference are handled with the standard token-classification pipeline with aggregation_strategy = “simple”, producing span-level entities with model-specific labels and logits. The generative models comprise four LLMs, Llama-3.2-3B^59^, Llama-3.2-3B-Instruct^60^, Llama-3.1-8B^61^ and Llama-3.1-8B-Instruct^62^. All Llama variants were executed locally via Ollama’s /api/chat endpoint, with the Llama-3.2 Instruct model quantized in Q6_K^63^ and Llama-3.1 Instruct model in Q4_0^64^ to ensure feasibility on consumer-grade hardware. For the Llamas, a common system prompt was used to enforce a strict JSON output schema, constrain the label set to the eight predefined entity types, and define the semantics of each category in PT–PT clinical language. Temperature was fixed at 0.0 to minimize stochastic variation, and the same two PT–PT few-shot examples were embedded in the user prompt for all models, teaching both the JSON format and the intended annotation behavior.

All experiments were run on a single laptop with a 12th Gen Intel Core i7-1255U CPU, integrated Intel UHD Graphics, 16 GB of RAM, and Windows 11. No discrete GPU was used, and models were executed sequentially to avoid contention for system resources. MediAlbertina was loaded via the Transformers library in Python, while the Llama models were served by a local Ollama instance. Each model processed the same 20 clinical reports, using the same input CSV and identifiers, and wrote its predictions to separate JSON files organized by batches in model-specific output directories. This ensured that differences in performance and runtime could be attributed to model architecture and size rather than to variations in data or infrastructure.

To enable a fair comparison across architectures, all outputs were normalized to a shared canonical format before evaluation. For MediAlbertina, raw label strings were mapped to the canonical tag set using a hand-crafted mapping that collapses morphological variants and synonyms. For the Llama runs, normalization operated at two levels: label strings were mapped through a similar canonicalization function, and entity spans were reconstructed when necessary. When models returned only text and type, approximate character offsets were recovered by searching the original note for the entity string, using a case- and accent-insensitive regular expression. All entities were then deduplicated within each document based on the triplet (start, end, type) to avoid double-counting overlapping predictions.

Evaluation followed a micro-averaged scheme over all documents. For each model, the gold entities were loaded into a dictionary keyed by document identifier, with each entry containing a list of pairs (type, normalized_text). Predicted entities were loaded from the model-specific output JSONs into the same structure. A “loose” matching criterion was adopted: a predicted entity was counted as a true positive if its type exactly matched the gold type and its normalized surface text either exactly matched or was a substring of the gold text (or vice-versa), reflecting the clinical practice of annotating short, semantically focused spans. Gold entities with no matching prediction were counted as false negatives; predicted entities not matched to any gold entity were counted as false positives. From these counts, micro-precision, micro-recall, and micro-F1 were computed across all documents.

In addition to the relaxed entity-level matching criterion, a strict character-span evaluation was performed to assess boundary-level NER performance. Under this stricter criterion, a predicted entity was counted as a true positive only when the entity type, start character offset, and end character offset matched a gold-standard entity in the same document. Before this evaluation, the same boundary standardization procedure was applied to both gold-standard and predicted entities by removing leading or trailing whitespace and terminal punctuation from the entity span. This standardization was applied uniformly across all models and did not modify the internal content of the entity mention. Predicted entities with incorrect labels, incorrect standardized offsets, or no corresponding gold-standard entity were counted as false positives, while gold entities without an exact standardized match were counted as false negatives. Strict precision, recall, and F1-score were then computed across all documents using the same micro-averaged aggregation scheme as the relaxed evaluation.

To account for uncertainty due to the small size of the proof-of-concept dataset, 95% bootstrap confidence intervals were computed for precision, recall, and F1-score under both the relaxed and strict evaluation settings. The bootstrap procedure was performed at the document level, using the 20 clinical reports as the resampling units. For each model and evaluation setting, true positives, false positives, and false negatives were first computed separately for each document using the corresponding matching criterion described above, either relaxed entity-level matching or strict character-span matching after boundary standardization. Then, 1000 bootstrap samples were generated by sampling 20 reports with replacement from the original set of 20 reports. For each bootstrap sample, precision, recall, and F1-score were recalculated by aggregating true positives, false positives, and false negatives across the resampled documents. The lower and upper bounds of the 95% confidence intervals were defined as the 2.5th and 97.5th percentiles of the resulting bootstrap metric distributions.

Wall-clock runtime for each model was measured using timestamps printed at the beginning and end of the extraction scripts, corresponding to the total time required to process all 20 clinical reports. These runtimes formed the basis for estimating energy consumption and CO₂ emissions. Because all experiments were executed locally on the same machine, the environmental impact was computed by converting total processing time into electrical energy consumption and subsequently into carbon emissions. The Intel Core i7-1255U was assumed to draw an average of 30 W under sustained computational load, a conservative approximation grounded in the official power envelope published by Intel^65^, which ranges from 15 W (Processor Base Power) to 55 W (Maximum Turbo Power).

Energy consumption was computed using:

$$Energy (kWh)=\frac{Power (W)\times Time (s)}{3600\times 1000}$$

where Power = 30 W and Time correspond to the measured runtime for each model.

To convert energy usage into CO₂ emissions, we applied the official Portuguese electricity carbon-intensity factor for Portugal Continental, 0.092 kgCO₂/kWh^66^, as published by the Portuguese Environment Agency (APA). The final emissions were therefore computed as:

$$CO_{2} (kg) = Energy (kWh) \times 0.092$$

To provide an intuitive reference for energy consumption, we estimated the equivalent diesel-equivalent consumption using the Gross Calorific Value of diesel (≈ 10.9 kWh/L)^67^. Diesel consumption was computed as:

$$\mathrm{D}\mathrm{i}\mathrm{e}\mathrm{s}\mathrm{e}\mathrm{l} (\mathrm{L})=\frac{{\mathrm{E}}_{\mathrm{k}\mathrm{W}\mathrm{h}}}{10.9}$$

These values should be interpreted as task-level estimates rather than direct power measurements. Energy consumption and CO₂ emissions were estimated from wall-clock runtime under an assumed average CPU power draw of 30 W, not measured using an external power meter or hardware-level energy profiler. Therefore, the reported values provide an approximate comparison of relative energy use for this specific workload, hardware configuration, and local execution setup. The diesel-equivalent conversion is included only as an intuitive reference and should not be interpreted as an independent environmental measurement.

#### Llama reproducibility detail

To improve reproducibility of the Llama-based experiments, all generative models were executed locally through Ollama using the HTTP /api/chat endpoint. The model name was controlled through the OLLAMA_MODEL environment variable, with the same extraction pipeline repeated for each evaluated Llama variant by changing only the model tag and the corresponding output directory. The evaluated Ollama model tags were llama3.2:3b, llama3.2:3b-instruct-q6_K, llama3.1:8b, and llama3.1:8b-instruct-q4_0. The corresponding quantization variants were the default Ollama quantization for llama3.2:3b and llama3.1:8b, Q6_K for llama3.2:3b-instruct-q6_K, and Q4_0 for llama3.1:8b-instruct-q4_0.

All models processed the same input CSV file containing the 20 Portuguese clinical reports, using DIARIO_CLINICO as the clinical-text column. Outputs were saved as batch-level JSON files in model-specific directories. The same system prompt, annotation schema, and two few-shot examples in European Portuguese were used across all Llama runs. The prompt instructed the model to behave as a precise clinical NER tagger for PT–PT clinical notes and to return only strict JSON following the schema { “entities”: [ {“text”: str, “type”: str, “start”: int, “end”: int, “score”: float} ] }. The allowed entity labels were: *Diagnostico*, *Sintoma*, *Medicamento*, *Dosagem*, *ProcedimentoMedico*, *SinalVital*, *Resultado*, and *Progresso*. The two few-shot examples illustrated the expected annotation of clinical progress, vital signs, results, medication, and dosage. The complete system prompt, user prompt template, few-shot examples, and fallback prompt are provided in Appendix 1 to support reproducibility.

For all Llama runs, temperature was fixed at 0.0. The decoding configuration used top_p = 0.1, repeat_penalty = 1.05, and num_predict = 768; format was set to json, and streaming was disabled (stream = False). No explicit context-length parameter was set in the extraction script; therefore, the default Ollama context-window configuration was used. Each document was processed sequentially, with a batch size of 8 and a maximum of three extraction attempts per document. If the initial attempts failed to return valid entities, a fallback prompt was used to reinforce extraction of vital signs/results, medication/dosage, and progress entities.

Model outputs were parsed automatically. First, the pipeline attempted to recover the JSON object by extracting the substring between the first opening brace and the last closing brace. The recovered string was then parsed using json.loads. Entity labels were normalized to the predefined canonical label set, and missing spans were reconstructed by searching the predicted mention in the original text using case-insensitive matching. Invalid labels were discarded, duplicate entities were removed based on (start, end, type), and only entities with valid character offsets within the original note were retained. If no valid JSON object or valid entity list could be recovered after the retry and fallback procedure, the output was treated as an empty prediction. No manual correction of model outputs was performed.

### Results

Table 4 summarizes the comparative performance of all five models on the Portuguese clinical NER task, including precision, recall, F1-score, and 95% bootstrap confidence intervals. The confidence intervals were added to reflect the uncertainty associated with the small proof-of-concept dataset of 20 clinical reports and 149 gold-standard entities. The domain-adapted encoder MediAlbertina achieved the highest overall performance, with an F1-score of 0.430 [0.344–0.539], substantially outperforming all Llama-based models, whose F1-scores ranged from 0.096 [0.041–0.189] to 0.123 [0.062–0.220]. MediAlbertina also obtained the highest recall, 0.651 [0.513–0.762], correctly recovering a larger proportion of the 149 gold-standard entities, although with moderate precision, 0.321 [0.250–0.439], due to over-generation. By contrast, all Llama variants produced markedly fewer entities than present in the gold annotations, resulting in consistently low recall values between 0.074 [0.029–0.178] and 0.087 [0.037–0.203]. Although Llama 3.2-3B-Instruct achieved the highest precision among the Llama models, 0.206 [0.136–0.333], its recall remained low, leading to an F1-score of 0.123 [0.062–0.220]. These results indicate that, under the tested prompting, quantization, and local execution configuration, the Llama models behaved conservatively, under-extracting entities and failing to match the coverage of the encoder-based clinical model.

**Table 4:** Comparative NER performance across models.

Framework	Gold Entities	Predicted Entities	Precision	Precision 95% CI	Recall	Recall 95% CI	F1	F1 95% CI	Time (s)	Estimated Energy (kWh)	Estimated CO₂ (g)	Diesel-equivalent (mL)
MediAlbertina	149	302	0.321	[0.250–0.439]	0.651	[0.513–0.762]	0.430	[0.344–0.539]	79	0.0007	0.06	0.06
Llama3.2 3B Instruct	149	63	0.206	[0.136–0.333]	0.087	[0.037–0.203]	0.123	[0.062–0.220]	4897	0.04	3.75	3.74
Llama3.1 8B	149	119	0.109	[0.057–0.181]	0.087	[0.037–0.191]	0.097	[0.045–0.178]	7476	0.06	5.73	5.72
Llama3.1 8B Instruct	149	120	0.108	[0.056–0.193]	0.087	[0.036–0.198]	0.097	[0.045–0.183]	6163	0.05	4.69	4.68
Llama3.2 3B	149	80	0.138	[0.069–0.250]	0.074	[0.029–0.178]	0.096	[0.041–0.189]	1791	0.01	1.37	1.37

To complement the relaxed entity-level evaluation, strict character-span performance was also assessed after boundary standardization. As shown in Table 5, strict scores were lower than relaxed scores across all models, reflecting the additional difficulty of recovering exact start and end character offsets. MediAlbertina remained the strongest model under this stricter criterion, achieving a strict F1-score of 0.302 [0.236–0.418], with strict precision of 0.225 [0.169–0.343] and strict recall of 0.456 [0.370–0.562]. The Llama-based models obtained substantially lower strict F1-scores, ranging from 0.017 [0.000–0.051] to 0.037 [0.009–0.086]. These results indicate that the relaxed metrics capture broader entity recovery, whereas the strict span-level metrics provide a more conservative estimate of exact boundary-level extraction.

**Table 5:** Strict character-span NER performance after boundary standardization.

Framework	TP	FP	FN	Strict Precision	P 95% CI	Strict Recall	R 95% CI	Strict F1	F1 95% CI
MediAlbertina	68	234	81	0.225	[0.169–0.343]	0.456	[0.370–0.562]	0.302	[0.236–0.418]
Llama3.2 3B Instruct	3	60	146	0.048	[0.000–0.109]	0.020	[0.000–0.060]	0.028	[0.000–0.069]
Llama3.1 8B	4	115	145	0.034	[0.008–0.076]	0.027	[0.005–0.075]	0.030	[0.007–0.073]
Llama3.1 8B Instruct	5	115	144	0.042	[0.011–0.088]	0.034	[0.008–0.090]	0.037	[0.009–0.086]
Llama3.2 3B	2	78	147	0.025	[0.000–0.065]	0.013	[0.000–0.047]	0.017	[0.000–0.051]

In terms of computational efficiency, large differences emerged between MediAlbertina and the Llama family. Processing the 20 clinical reports required 79 s for MediAlbertina, compared with 1 791 s for Llama 3.2-3B, 4 897 s for Llama 3.2-3B-Instruct, 6 163 s for Llama 3.1-8B-Instruct, and 7 476 s for Llama 3.1-8B. This corresponds to speed factors of approximately 23 × to 95 × slower than the encoder baseline, despite the Llama models delivering substantially lower F1-scores. When energy consumption is taken into account, the contrast is equally pronounced: estimated energy usage ranged from 0.00066 kWh for MediAlbertina to 0.0149 kWh for Llama 3.2-3B, 0.0408 kWh for Llama 3.2-3B-Instruct, 0.051 kWh for Llama 3.1-8B-Instruct, and 0.0623 kWh for Llama 3.1-8B. After conversion using the Portuguese continental grid emission factor (0.092 kgCO₂/kWh), this yields an approximate carbon footprint of 0.0607 g CO₂ for MediAlbertina, compared with 1.37 g for Llama 3.2-3B, 3.75 g for Llama 3.2-3B-Instruct, 4.69 g for Llama 3.1-8B-Instruct, and 5.73 g for Llama 3.1-8B over the same workload. When expressed in diesel-equivalent terms, this corresponds to approximately 0.061 mL for MediAlbertina, 1.37 mL for Llama 3.2-3B, 3.74 mL for Llama 3.2-3B-Instruct, 4.68 mL for Llama 3.1-8B-Instruct, and 5.72 mL for Llama 3.1-8B. The diesel-equivalent values are reported only as an intuitive reference and are not treated as central scientific outcomes.

Taken together, these findings show that, in this specific local benchmark, the specialized encoder-based LM achieved better NER performance while requiring substantially lower runtime and lower estimated task-level energy consumption and CO₂ emissions than the evaluated quantized Llama models. The Llama models therefore exhibited an unfavorable trade-off between accuracy, latency, and estimated task-level environmental cost under the tested workload, hardware, quantization, and prompting configuration.

Error analysis showed that the Llama-based models mainly failed through under-extraction, producing substantially fewer entities than the gold standard. False negatives were particularly relevant for short clinical mentions and repeated structured elements such as vital signs, results, medication/dosage pairs, and progress expressions. Invalid or partial JSON outputs were handled automatically through the predefined parsing and fallback procedure, when no valid entity list could be recovered, the prediction was treated as empty. This behavior contributed to the low recall observed across all Llama variants.

## Discussion

### Performance vs. efficiency trade-off

A central finding of both the recent literature and the present case study is an asymmetric trade-off between extraction quality and computational burden: while performance gains from scaling to LLMs are often incremental, increases in cost (parameters, memory, latency, and energy) tend to be superlinear. In clinical NER specifically, multiple comparative studies report that LLM-based approaches can occasionally yield small marginal improvements in F1-score relative to smaller or encoder-based baselines, but only under carefully optimized prompting, fine-tuning, or retrieval settings^39^. These limited gains must be weighed against the substantially higher resource requirements typical of LLM inference and adaptation, including increased VRAM demands, slower throughput, and higher monetary cost when using cloud APIs^52^.

The results of our Portuguese clinical NER case study show an even sharper imbalance. The domain-adapted encoder baseline (MediAlbertina-1.5B) achieved a micro-F1 of 0.430, whereas all tested Llama models remained between 0.096 and 0.123, representing an absolute decrease of ~ 0.31–0.33 F1 rather than an improvement. This performance gap is explained primarily by recall: the Llama models systematically under-extracted entities, yielding consistently low recall (0.074–0.087) despite moderate precision in some configurations. In contrast, MediAlbertina recovered a substantially larger fraction of gold entities (recall = 0.651), albeit with over-generation and reduced precision. These findings align with prior observations that generative LLMs often struggle with token-level coverage and exhaustive span recovery in label-dense clinical documents, tending toward conservative extraction behavior that depresses recall^18^.

Beyond extraction quality, the present findings further corroborate literature reports indicating that the computational cost of LLM-based NER may involve higher computational cost relative to performance gains. Prior studies consistently show that inference with instruction-tuned LLMs entails substantially higher latency and memory requirements than encoder-only models, often requiring tens of gigabytes of GPU VRAM and exhibiting markedly reduced throughput in token-level extraction tasks^15,49^. This pattern was clearly reflected in our experiments: MediAlbertina processed the full dataset in 79 s, whereas the Llama variants required between 1791 and 7476 s, corresponding to a slowdown of approximately 23 × to 95 × . These results align with earlier evidence that LLM inference costs scale superlinearly with model size and prompt complexity, particularly in locally executed or resource-constrained settings^21,49^.

In this task-level estimation, the increase in latency corresponded to higher estimated energy consumption and CO₂ emissions, reinforcing concerns raised in recent efficiency- and sustainability-oriented studies^20,21^. While prior work often discusses the environmental footprint of large models at an abstract or infrastructure level, the present case study provides task-level estimates suggesting that the evaluated Llama models incurred one to two orders of magnitude higher estimated energy use than MediAlbertina under this workload and hardware setup, even when executed on identical hardware. When translated into diesel-equivalent terms, the same workload corresponds to approximately 0.061 mL for MediAlbertina and between 1.37 and 5.72 mL for the Llama variants, further illustrating the magnitude of the efficiency gap in tangible fuel terms. Taken together, these results are consistent with existing literature suggesting that, in structured clinical extraction scenarios similar to the present setup, prompted LLMs may not deliver proportional accuracy gains relative to their computational cost, further limiting their suitability for high-volume, routine information extraction workflows^18^.

### Applicability and scalability

Beyond raw performance, the literature consistently emphasizes that the practical applicability of LMs is strongly shaped by deployment constraints rather than theoretical capabilities^18^. The reviewed literature suggests that encoder-based LMs and SLMs are better suited for integration into structured, repetitive, and high-volume clinical workflows, where determinism, high recall, and predictable latency are critical requirements^15,18,35^. In tasks such as clinical NER, structured information extraction, and rule-governed question answering, the objective is not abstract reasoning but exhaustive and stable coverage of predefined entity types^18^. Domain-adapted encoder models may be advantageous under these conditions, as their token-level predictions are optimized through supervised fine-tuning and remain consistent across documents, potentially supporting more predictable processing in local or resource-constrained settings^17,18,47^.

In contrast, LLMs are consistently reported to be more advantageous in scenarios requiring zero-shot generalization, cross-domain reasoning, or open-ended text generation, including summarization, synthesis, and exploratory clinical decision support^1,13,48^. However, the same characteristics that make LLMs effective in these settings, generative flexibility, probabilistic reasoning, and context-sensitive abstraction, , may become liabilities in structured extraction tasks^18^. The literature shows that LLMs tend to trade recall for precision, exhibit variable output formats, and incur substantially higher inference latency, rendering them inefficient for repetitive, schema-driven tasks such as NER or routine clinical QA^15,18,35^. Moreover, their frequent reliance on cloud-based execution introduces additional challenges related to data governance, privacy, and scalability, particularly in healthcare environments where data locality and regulatory compliance constrain deployment choices^17,18,50^. As a result, while LLMs occupy an important role in zero-shot and creative applications, existing evidence supports the continued preference for encoder-based LMs and SLMs in production-grade clinical extraction systems, where scalability is defined not by model size but by reliability, efficiency, and deployment feasibility.

### Towards hybrid and efficient solutions

Rather than reinforcing a binary opposition between encoder-based LMs and large generative LLMs, the recent literature increasingly converges on hybrid and efficiency-oriented design strategies that seek to combine the strengths of both model classes^43^. Parameter-efficient adaptation techniques, such as LoRA, QLoRA, adapter tuning, and quantization-aware fine-tuning, exemplify this shift by enabling large models to be specialized for domain-specific tasks while updating only a small fraction of parameters^1^. These methods substantially reduce VRAM requirements and training cost, demonstrating that much of the representational and reasoning capacity of LLMs can be preserved without incurring the full computational burden of dense fine-tuning^9^. In this sense, scale is no longer treated as an all-or-nothing choice, but as a resource that can be selectively compressed and deployed where it provides tangible benefit.

Beyond fine-tuning, knowledge distillation and selective invocation strategies offer a system-level resolution to the performance–efficiency tension observed in this study. In distillation-based approaches, LLMs act as teachers that transfer domain knowledge, reasoning patterns, or synthetic supervision to smaller encoder-based models, confining the cost of large models to offline training while maintaining lightweight, deterministic inference at deployment time^54^. Complementarily, hybrid pipelines such as filter–then–rerank architectures allocate computational effort adaptively: encoder-based LMs or SLMs handle the majority of routine, high-confidence cases, while LLMs are invoked only for a small subset of difficult or ambiguous instances^43^. This paradigm is consistent with the empirical findings of the present case study, where the evaluated Llama models were less efficient and recall-limited when used end-to-end. The results from our study support a more cautious unifying conclusion: clinical information extraction may benefit less from the routine use of ever-larger standalone models and more from the principled allocation of model capacity across modular, hybrid systems. In such systems, larger models can be used sparingly and strategically, particularly in cases where they provide measurable benefits over smaller or domain-adapted models^15^.

### Limitations

As a proof-of-concept study, the findings should be interpreted within the scope of the experimental setup. The empirical benchmark was conducted on a limited dataset of Portuguese clinical reports containing 149 gold-standard entities. As a result, the findings provide an initial indication of model behavior in this specific PT–PT clinical NER setting, but broader generalization to other languages, hospitals, clinical specialties, document types, or larger healthcare corpora requires external validation. Nevertheless, the aim of this case study was not to establish a definitive benchmark for all clinical NLP settings, but to compare extraction performance, runtime, and task-level sustainability indicators under a controlled local-deployment scenario. In this sense, the results can help inform future methodological choices when selecting between domain-adapted encoder models and locally prompted quantized LLMs for structured clinical information extraction.

The gold-standard annotations were produced by an oncology specialist affiliated with the Champalimaud Foundation, which provided clinical expertise to the annotation process. However, because only one specialist annotator was involved, inter-annotator agreement metrics could not be computed, and no formal adjudication process between independent annotators was performed.

All experiments were executed locally on a single CPU-only laptop without a discrete GPU. This setup was intentionally aligned with a resource-constrained local deployment scenario, but it does not represent all possible infrastructure settings. Runtime and feasibility may differ on GPU-equipped workstations, hospital servers, cloud platforms, or optimized inference engines. In addition, the evaluated Llama models were quantized to enable local execution, which improves feasibility but may affect performance compared with non-quantized or differently quantized versions.

## Conclusions and future directions

The rapid adoption of LLMs in clinical and biomedical NLP has led to a widespread perception that scale alone is the primary driver of progress. The findings of this study challenge this assumption by showing that, for structured and repetitive tasks such as clinical NER, classical and domain-adapted LMs remain not only competitive, but often superior when evaluated under realistic deployment constraints. While LLMs offer undeniable advantages in zero-shot reasoning, generative flexibility, and cross-domain abstraction, the present results demonstrate that these strengths do not systematically translate into improved token-level extraction performance, particularly when recall, latency, energy consumption, and deployment feasibility are jointly considered.

All research objectives defined in this study were addressed through a combination of scoping literature review and empirical evaluation. O1 (Performance) was addressed through the comparative analysis of encoder-based LMs, SLMs, and LLMs in the literature (Sect. “Language Models” and “Large language models (LLMs)”) and empirically validated in the proof-of-concept study (Sect. “Results”), where the domain-adapted encoder MediAlbertina achieved the highest F1-score (0.430), substantially outperforming the Llama-based models. O2 (Efficiency) was examined in Sect. “Efficiency in model scaling” and quantified in Sect. “Results”, revealing that LLM inference was 23–95 × slower than the encoder baseline. O3 (Environmental Impact) was addressed through the task-level estimation of energy consumption and CO₂ emissions in Sects. “Methodology” and “Results”, showing one to two orders of magnitude higher environmental footprint for LLMs. O4 (Deployment Feasibility) and O5 (Privacy and Governance) were analyzed through both the literature (Sects “Efficiency in model scaling” and “Empirical comparison of SLMs, LMs, and LLMs”) and the local execution of all models on on-premise hardware (Sect. “Methodology”), highlighting the practical advantages of smaller models in regulated clinical environments. Finally, O6 (Reliability) was addressed through the literature-based discussion of hallucination risks and the empirical analysis of boundary errors, false positives, invalid outputs, and systematic under-extraction.

Taken together, these results provide a clear and empirically grounded answer to the RQ within the scope of the evaluated setting: In the evaluated Portuguese clinical NER benchmark, the domain-adapted MediAlbertina model was more suitable than the locally executed Llama models when extraction performance and local-deployment efficiency were jointly considered. In this setting, MediAlbertina achieved higher recall and F1-score while simultaneously requiring substantially lower runtime, estimated energy consumption, and estimated CO₂ emissions. These findings support the use of smaller, domain-adapted encoder models for structured clinical NER tasks, particularly in local and resource-constrained settings, while larger and externally validated benchmarks are still needed before generalizing to other languages, clinical corpora, model families, or deployment environments.

From a broader perspective, the state of the art increasingly points toward hybrid and efficiency-oriented solutions rather than unbounded model scaling. Recent advances in parameter-efficient fine-tuning, quantization, knowledge distillation, and selective invocation architectures suggest that the future of clinical NLP lies in better allocation of intelligence, not larger standalone models. In this context, the present work contributes empirical evidence that supports a shift from model-centric to system-centric design: encoder-based LMs may serve as the backbone of high-volume clinical extraction systems, while LLMs are best positioned as complementary components, selectively deployed for complex reasoning, ambiguity resolution, or offline knowledge transfer. This perspective not only aligns with current efficiency and sustainability concerns but also provides a pragmatic roadmap for deploying NLP systems that are accurate, scalable, and compatible with the operational realities of modern healthcare environments.

## Supplementary Information

Below is the link to the electronic supplementary material.

Supplementary Material 1.

## Data Availability

The datasets analysed during the current study are not publicly available because they contain sensitive clinical data and are subject to institutional and legal restrictions regarding patient privacy. Access to de-identified data may be considered by the corresponding author on reasonable request and subject to approval by the relevant institution and ethics/regulatory requirements.
